# Cross-sector collaborations in Aboriginal and Torres Strait Islander childhood disability: a systematic integrative review and theory-based synthesis

**DOI:** 10.1186/s12939-014-0126-y

**Published:** 2014-12-18

**Authors:** Anna Green, Michelle DiGiacomo, Tim Luckett, Penelope Abbott, Patricia Mary Davidson, Joanne Delaney, Patricia Delaney

**Affiliations:** Center for Cardiovascular and Chronic Care, Faculty of Health, University of Technology, Sydney, PO Box 123, Broadway, NSW 2007 Australia; University of Western Sydney, Locked Bag 1797, Penrith, NSW 1797 Australia; School of Nursing, Johns Hopkins University, 525 N. Wolfe Street, Baltimore, MD 21205 USA; Aboriginal Medical Service Western Sydney, PO Box 3160, Mt Druitt, NSW 2770 Australia

**Keywords:** Aboriginal and Torres Strait Islander, Childhood, Disability, Collaboration, Inter-sector, Intra-sector

## Abstract

**Introduction:**

Aboriginal and Torres Strait Islander children in Australia experience a higher prevalence of disability and socio-economic disadvantage than other Australian children. Early intervention is vital for improved health outcomes, but complex and fragmented service provision impedes access. There have been international and national policy shifts towards inter-sector collaborative responses to disability, but more needs to be known about how collaboration works in practice.

**Methods:**

A systematic integrative literature review using a narrative synthesis of peer-reviewed and grey literature was undertaken to describe components of inter- and intra-sector collaborations among services to Aboriginal and Torres Strait Islander children with a disability and their families. The findings were synthesized using the conceptual model of the ecological framework.

**Results:**

Thirteen articles published in a peer-reviewed journal and 18 articles from the grey literature met inclusion criteria. Important factors in inter- and intra-sector collaborations identified included: structure of government departments and agencies, and policies at the macro- (government) system level; communication, financial and human resources, and service delivery setting at the exo- (organizational) system level; and relationships and inter- and intra-professional learning at the meso- (provider) system level.

**Conclusions:**

The policy shift towards inter-sector collaborative approaches represents an opportunity for the health, education and social service sectors and their providers to work collaboratively in innovative ways to improve service access for Aboriginal and Torres Strait Islander children with a disability and their families. The findings of this review depict a national snapshot of collaboration, but as each community is unique, further research into collaboration within local contexts is required to ensure collaborative solutions to improve service access are responsive to local needs and sustainable.

## Introduction

In contrast to other countries, the Australian population has access to a first-class universal healthcare system and is relatively healthy [1]. Aboriginal and Torres Strait Islander peoples are an exception to this rule. The gap in health outcomes and life expectancy between Aboriginal and Torres Strait Islander peoples and other Australians has been widely reported [1–3]. The rate of death for Aboriginal and Torres Strait Islander children is more than twice that for other children [2]. This disparity in health outcomes extends to disability [4]. Increasingly there is recognition of the importance of the social determinants of health and of health as a human right.

### Social determinants of health and human rights

Although there are social gradients in the incidence of disability, it is reported that little attention has been paid to research on the social determinants of health in disability policy [5]. Policy has the potential to act as a structural determinant of health [6]. The Australian Human Rights Commission has drawn attention to a number of human rights violations faced by Aboriginal and Torres Strait Islander persons with a disability. These include individual rights to health and education that are impacted by the high levels of socio-economic disadvantage [7]. The link between disability and poverty is bi-directional [8]. In the United States and Canada, indigenous populations also experience the negative impact of socio-economic disadvantage on service access [9–11]. Racism is another key social determinant of health that negatively impacts service access [12]. Experiences of direct and indirect racism have been linked to distrust of mainstream organizations and providers [2,13].

#### Health disparities in childhood disability

Aboriginal and Torres Strait Islander children experience a higher prevalence of disability than other children [4]. They encounter higher rates of hearing loss [14,15] which has been linked to the high prevalence of middle ear diseases such as otitis media (OM). Rates of OM experienced by Aboriginal and Torres Strait Islander children are among the highest in the world, similar to those in low income countries and at a level classified by the World Health Organization (WHO) as a massive public health problem [2,16,17]. OM is also experienced for longer and more persistent periods by Aboriginal and Torres Strait Islander children (32 months compared with 3 months for other children) [18,19]. Aboriginal and Torres Strait Islander children have also been found to have a significantly higher prevalence of communication disorders [20] and are 1.3 times as likely to require assistance with self-care, mobility or communication than other children [21]. Such disparity is also evident in developmental delay [22,23]. Early intervention is vital as high rates of disability can negatively impact education, speech, language and social development, and employment outcomes [13,14,17,19,24–26]. It is also acknowledged that intervening at the early stages of childhood development is more cost-effective than intervening later in life [27].

#### Social determinants of health and Aboriginal and Torres Strait Islander childhood disability

Aboriginal and Torres Strait Islander children not only experience a higher prevalence of disability but are also disproportionately affected by socio-economic disadvantage [2]. Almost half of Aboriginal and Torres Strait Islander households are in the lowest income group and are 4 times less likely to be in the highest group than other Australians [2]. Socio-economic disadvantage directly impacts disability for Aboriginal and Torres Strait Islander children [25] who are more likely to experience negative developmental outcomes from disabilities like OM-related hearing loss due to social determinants of health [18]. Addressing the influence of social determinants of health on Aboriginal and Torres Strait Islander childhood disability requires a shift in thinking as they are often considered indirect to the traditional responsibilities of health, education, and social service sectors [25,28,29].

### Barriers to service access

Aboriginal and Torres Strait Islander children with a disability and their families face many barriers to service access [25]. A key barrier is the confusion caused by complex and fragmented service provision across government departments and agencies working in professional silos [30,31]. This lack of integration is often described by a silo approach. A silo refers to systems and processes that operate in isolation from each other.

### Policy response to improve service access

The need for holistic and collaborative responses to disability is recognized internationally [8]. The World Report on Disability identifies that policies within health, education and social service sectors all impact on disability outcomes [8]. Nationally, the Australian Government’s “Close the Gap” campaign to reduce Aboriginal and Torres Strait Islander disadvantage advocates the need for collaboration across all sectors and levels of Government for effective service coordination [32]. The national policy direction towards collaboration and whole-of-government approaches is reflected in a number of disability-specific policies and strategic frameworks [3,33–36].

Little is known about Aboriginal and Torres Strait Islander children with a disability [4]. Despite the policy push towards collaboration, there has been no systematic attempt to elucidate how collaboration works in practice across and within sectors involved in service provision. Therefore, the current authors set out to answer the question: What are the important components involved in inter- and intra-sector collaboration in Aboriginal and Torres Strait Islander childhood disability? Understanding these components will be essential in improving service provision and access for Aboriginal and Torres Strait Islander children with a disability and their families.

## Methods

We conducted an integrative literature review using a systematic approach to identify components of collaboration guided by an investigator-developed protocol.

### Eligibility criteria

Disability is a complex concept with no universally agreed definition [8]. For the purposes of this review, disability refers to long-term physical, mental, intellectual or sensory impairments that, interacting with environmental and attitudinal barriers, hinder full and effective participation in society on an equal basis with others [37].

Included articles focused on Aboriginal and Torres Strait Islander children with a disability and/or their families/carers, or providers of services to this population (eg from the health, education and social service sectors), and include reference to collaboration or interaction within or across two or more providers/sectors. We included articles in the English language specifically addressing Australian issues. No publication date limits were imposed and all study designs were included be they quantitative, qualitative or mixed methods. Commentaries were also included. Articles were included regardless of whether they were published in peer-reviewed journals or grey literature. Articles were excluded if their sole focus was on adolescent or adult disability or a population other than Aboriginal and Torres Strait Islander peoples.

### Search strategy

**Figure 1:**
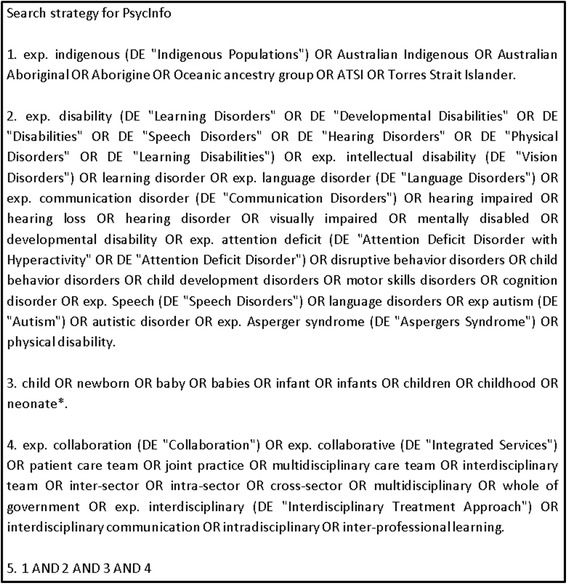
Electronic database search strategy example*. *Search terms varied slightly for each database.

A systematic electronic database search strategy using Boolean terms was developed in collaboration with a health librarian. Search terms were Medical Subject Headings (MeSH) terms and keywords including derivatives of the key terms ‘collaboration’, ‘child’, ‘disability’ and ‘indigenous’ (see Figure 1 for an example). The grey literature was searched using variations of the key search terms from each of these groupings.

### Information sources

A systematic search of health, education, social science, multidisciplinary and indigenous electronic databases was conducted to identify articles published in peer-reviewed journals. The electronic databases Cumulative Index to Nursing and Allied Health Literature (CINAHL), EMBASE, PsycInfo, Medline, Education Resources Information Center (ERIC), Social Services Abstracts, Sociological Abstracts, Academic Search Complete, Health Collections (Informit), Indigenous Studies Bibliography (AIATSIS), Australian Public Affairs Information Service (APAIS), Australian Public Affairs Information Service - Health (APAIS-health), Aboriginal and Torres Strait Islander Health (A&TSIhealth), Health & Society, Multicultural Australia and Immigration Studies - Aboriginal and Torres Strait Islander Subset (MAIS-ATSIS), Rural and Remote Health Database (RURAL), Australian Indigenous HealthInfoNet and Google Scholar search engine were searched from 13th – 14th May 2014. Reference lists were also searched for relevant articles.

Grey literature was identified through a search of websites of Aboriginal and Torres Strait Islander and disability representative organizations, the National Disability Organisations’ Clearinghouse, Trove theses database, and Mednar from 23rd May – 4th June 2014. Grey literature identified during the search for articles published in peer-reviewed journals was also reviewed.

### Study selection

Returned articles published in peer-reviewed journals were imported into EndNote software. One hundred articles were assessed against eligibility criteria independently by two researchers (AG and MD). Any inconsistencies were discussed until consensus was reached. One researcher (AG) assessed the remaining articles.

### Data collection

Data were extracted from the original text of included articles by AG into an a priori designed electronic spreadsheet. Data items included the setting, design, disability/impairment, population, aims, and methods. Data items specific to collaboration were extracted and grouped according to the discipline of providers involved in collaboration, collaborative models, components of collaboration, and key conclusions or recommendations.

### Evaluation and analysis

**Figure 2:**
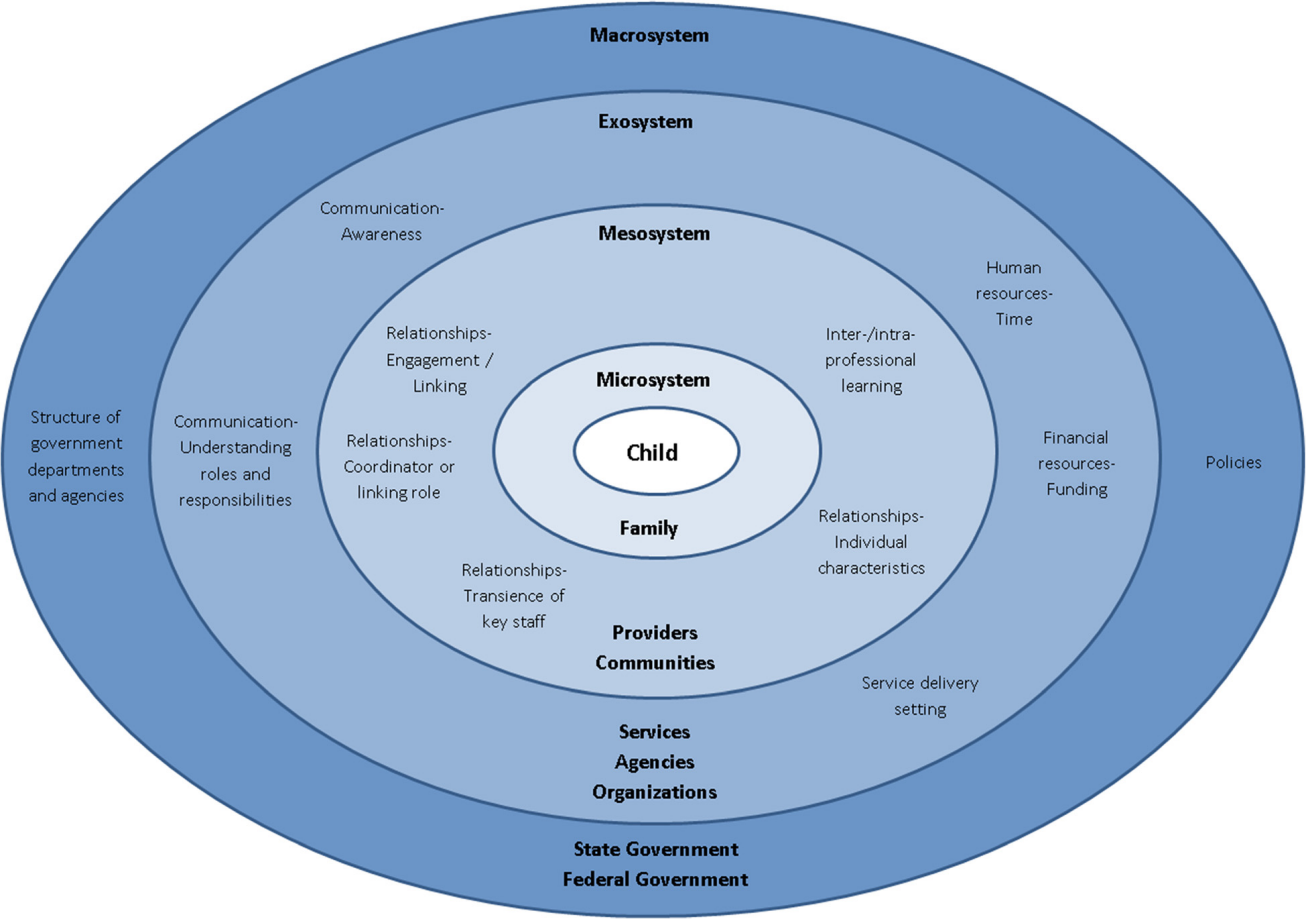
**Factors of inter- and intra-sector collaboration in Aboriginal and Torres Strait Islander childhood disability.** *Source:* Adapted from the Australian Institute of Health and Welfare 2012 [45].

Quality appraisal of the articles published in a peer-reviewed journal was conducted as part of a systematic approach to provide an overview of quality, but was not given weighting in the analysis and synthesis of data due to the lack of formal methods for this in integrative reviews. Quality appraisal of all included articles published in a peer-reviewed journal was conducted independently by two researchers (AG-MD or AG-TL) who met to establish agreement on the final rating. Any disagreements were resolved through discussion. The following critical appraisal tools were used: criteria for assessing qualitative literature [38], the STrengthening the Reporting of OBservational studies in Epidemiology (STROBE) checklist [39], the Transparent Reporting of Evaluations with Nonrandomized Designs (TREND) checklist [40], the Mixed Methods Appraisal Tool (MMAT) [41], and the Measurement Tool to Assess Systematic Reviews (AMSTAR) checklist [42] to assess qualitative, observational, intervention, mixed methods, and review studies, respectively. All included articles were evaluated using the Level of Evidence ranking system by MeInyk and Fineout-Overholt [43]. Data analysis was guided by the narrative synthesis approach by Popay et al. [44]. After developing the preliminary synthesis of findings we searched for a conceptual model. The model needed to provide a holistic framework centered on the child and their family that encompassed the different system levels of collaboration and how they interact with one another. An adaptation [45] of Bronfenbrenner’s ecological model for child development [46] represented a conceptual model in which the relationships in the data could be explored at the macro- (government), exo- (organizational) and meso- (provider) system levels (see Figure 2). The ecological model has previously been referenced in the context of addressing factors influencing equitable service access for underserved populations with a communication disability [47]. To our knowledge, it hasn’t before been applied specifically to service access issues in Aboriginal and Torres Strait Islander childhood disability. This organizing framework reflects factors that interact to achieve a desired outcome and also the impact of social interaction. Addressing each element discretely without considering the interdependency of elements is unlikely to achieve desirable outcomes.

## Results

**Figure 3:**
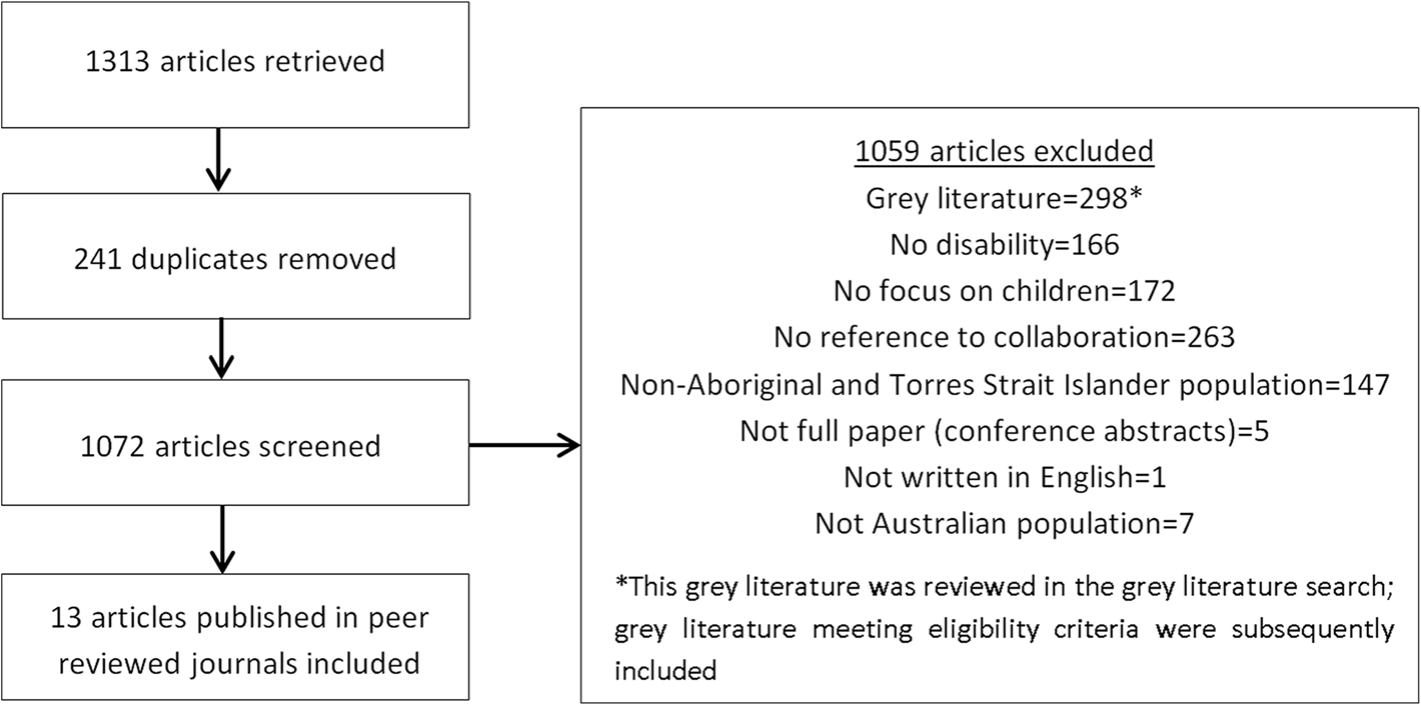
**PRISMA flowchart of search for peer-reviewed journal articles.**

The database search and peer-reviewed article selection is depicted in Figure 3. Thirteen peer-reviewed articles met inclusion criteria. The majority of studies were qualitative (n = 5) (Table 1) followed by discussion papers (n = 3) (Table 2), observational (n = 2) (Table 3), intervention (n = 1) (Table 4), mixed methods (n = 1) (Table 5) and literature review (n = 1) (Table 6). The grey literature search retrieved 18 articles that met the inclusion criteria (Table 7). In total, 31 articles were included in the review.

**Table 1:** **Qualitative studies**

**First Author (year)**	**Disability/Impairment**	**Design**	**Level of evidence**	**Population**	**Setting**	**Aims**	**Methods**	**Model involving collaboration**
Davidson, B. (2013) [72]	Communication	Qualitative	VI	Aboriginal and Torres Strait Islander children	Aboriginal and Torres Strait Islander Independent Community School; Urban; Queensland	To raise awareness through lessons learned from an inter-professional clinic.	Survey with open ended questions of university students on placement; Informal feedback from teachers	Yes
DiGiacomo, M. (2) (2013) [53]	General disability	Qualitative	VI	17 government and non-government health and social service providers; 5 carers	Aboriginal Community Controlled Health Service; Urban; New South Wales	To determine the elements involved in service access for urban Aboriginal children with a disability.	Community forums using focus group methods	No
McSwan, D. (2001)* [68]	Hearing	Evaluation	VI	Indigenous children from 3 rural/remote schools	Rural/remote communities	Report on a project aiming to develop a whole of community approach to the impact of OM on learning.	Questionnaires and interviews from persons involved in program implementation	Yes
Nelson, A. (2004, 2007) [66,67]	Physical; Developmental; Learning; General disability	Qualitative	VI	43 Indigenous students	Primary schools and preschools; Urban; Queensland	To evaluate a pilot project and explore the elements of a culturally and socially appropriate occupational therapy service.	Focus groups and interviews with teachers and parents; Semi-structured qualitative survey	Yes

*Reports on the same study as the included grey literature report: McSwan, D. et al. (2001) Report: A Whole Community Approach to Otitis Media - reducing its incidence and effects. Townsville: Rural Education, Research & Development Centre, James Cook University.

**Table 2:** **Discussion papers**

**First Author (year)**	**Disability/Impairment**	**Design**	**Level of evidence**	**Population**	**Setting**	**Aims**	**Methods**	**Model involving collaboration**
Aldred, R. (2002) [20]	Development	Discussion paper	VI	Aboriginal children under 5 years old	Urban; Queensland	To explain how the development of a speech pathology position in an Indigenous Hearing Health Service sought to address service access issues.	Author observation and reflections	No
Clarke, K. (2013) [48]	Development; Learning	Discussion paper; Model	VI	Rural and remote Aboriginal children	Rural and remote communities	To present the SpICE Model as part of the solution to the promotion of child wellbeing.	Author observation and reflections	Yes
Kirkham, L-A. (2010) [59]	Hearing	Discussion paper; Conference report	VII	Indigenous children	Australia	To share findings from the Australian Otitis Media workshop.	Author observations and reflections	No

**Table 3:** **Observational studies**

**First Author (year)**	**Disability/Impairment**	**Design**	**Level of evidence**	**STROBE score**	**Population**	**Setting**	**Aims**	**Methods**	**Model involving collaboration**
Adams, K. (2004) [65]	Hearing	Observational; quantitative	IV	16	Indigenous children aged 0–11 years old	Gippsland Region; Victoria	To describe the Gippsland Indigenous Hearing Health Program and evaluation results.	Analysis of ear screening outcomes and management	Yes
Smith, A. (2012)* [74]	Hearing	Observational; longitudinal study	IV	16	Indigenous children from 21 schools	Remote Aboriginal community; Central Queensland	To observe the outcomes of the ear screening service in the first 3 years.	Retrospective review of service activity	Yes

*Study is looking at the same service as Elliott, G. (2010) [70].

**Table 4:** **Intervention study**

**First Author (year)**	**Disability/Impairment**	**Design**	**Level of evidence**	**TREND score**	**Population**	**Setting**	**Aims**	**Methods**	**Intervention type**	**Model involving collaboration**
Elliott, G. (2010)* [70]	Hearing; vision	Intervention	IV	15	442 Aboriginal and Torres Strait Islander children, from 0–6 years old	South Burnett region; Queensland	Feasibility of integrating a mobile telehealth-enabled screening service with existing community health services.	Feasibility determined by the number of consenting children, referral rate, and three-point categorical scale rating the quality of screening images	Mobile telehealth screening service	Yes
**First Author (year)**	**Intervention recruitment**	**Intervention control Group**		**Intervention content/Components**		**Intervention Duration**		**Intervention Evaluation**		
Elliott, G. (2010) [70]	Schools disseminated consent forms and information sheets; children with parental consent were screened	None		An Aboriginal health worker coordinated a mobile health-screening service which was taken to daycare centers and primary schools. Assessment results were put into a secure database and referrals for review and management were made to local health services and tele-otology clinics.		6 months		Community acceptance, the practical feasibility of presenting diagnostic information for online consultations, and integration with existing community services were evaluated for feasibility.		

*Study is looking at the same service as Smith, A. (2012).

**Table 5:** **Mixed method study**

**First Author (year)**	**Disability/Impairment**	**Design**	**Level of evidence**	**MMAT score**	**Population**	**Setting**	**Aims**	**Methods**	**Model involving collaboration**
Raman, S. (2011) [63]	Developmental	Mixed methods (quantitative/qualitative)	VI	*50% **75% ***50%	Aboriginal children in out-of-home care	Urban; New South Wales	Evaluation of the multidisciplinary KARI clinic and its outcomes.	Semi-structured interviews; Review of clinical data collected on the first 100 children seen by the clinic	Yes

*Qualitative component; **Quantitative component; ***Mixed method component.

**Table 6:** **Literature Review**

**First Author (year)**	**Disability/Impairment**	**Design**	**Level of evidence**	**AMSTAR score**	**Population**	**Setting**	**Aims**	**Methods**	**Model involving collaboration**
DiGiacomo, M (1) (2013) [51]	General disability	Integrative literature review	V	7	Aboriginal and Torres Strait Islander children	Australia	To ascertain the elements that impact on access to support and management, diagnosis and prevention.	Integrative review using systematic methods with a narrative synthesis	No

**Table 7:** **Grey literature**

**Citation**	**Disability/Impairment**	**Design**	**Level of evidence**	**Focus/Setting**	**Model involving collaboration**
(2006). Australian Indigenous Ear*InfoNet* and *InfoNetwork. Aboriginal and Islander Health Worker Journal*, July/August 30(3). [61]	Hearing	Content overview	VII	Aboriginal children; To provide information on the Indigenous Ear*lnfoNet* web resource to support an Indigenous Ear*InfoNetwork*	No
(2013). Otitis media: helping to close the gap in Indigenous Australia. *Medicus (Nedlands, WA)*, 53(2). [26]	Hearing	Description of the Earbus program	VII	Description of the Telethon Speech Hearing Centre for Children’s Earbus Program in Western Australia which provides ear health checks to Aboriginal and Torres Strait Islander children.	Yes
ARTD Consultants (2008). Evaluation of the Aboriginal otitis media screening program: Final Report. Sydney: NSW Health. [55]	Hearing	Mixed methods; Semi-structured interviews; Case studies; Analysis of screening data	VI	The Aboriginal Otitis Media Screening Program provides free screening to Aboriginal children between 0–6 years old. The aim of the evaluation was to gather information on the program’s appropriateness and inform future policy directions.	No
Australian Institute of Health and Welfare (2014). Stronger Futures in the Northern Territory: Hearing Health Services 2012–2013. Canberra: Australian Institute of Health and Welfare. [24]	Hearing	Evaluation of data collected by relevant health professionals on service provided and demographic characteristics of the children	VI	This report provides data on the Northern Territory Child Hearing Health Coordinator (CHHC) initiative.	Yes
Burns, J. & Thomson, N. (2013). Review of ear health and hearing among Indigenous Australians. Western Australia: Australian Indigenous Health*InfoNet*. [49]	Hearing	Narrative literature review	VII	This review provides an overview of the ear health and hearing of Aboriginal and Torres Strait Islander peoples to support the development of future policies and programs.	No
Burrow, S., Galloway, A., & Weissofner, N. (2009). Review of educational and other approaches to hearing loss among Indigenous people. Western Australia: Australian Indigenous Health*InfoNet*. [58]	Hearing	Literature review	VII	Summary of the literature on educational and other approaches to hearing loss in Indigenous populations.	No
Burton, J. (2012) Opening Doors Through Partnerships: Practical approaches to developing genuine partnerships that address Aboriginal and Torres Strait Islander community needs April 2012. Victoria: SNAICC. [64]	General disability	Case study analysis approach of interviews; Reports on 9 case studies	VI	Explores the steps mainstream service providers, Aboriginal Community Controlled Organisations and government can take to develop and support partnerships to increase the quality and choice of culturally appropriate services.	No
Gilroy, J. (2012) The participation of Aboriginal people with a disability in disability services in New South Wales, Australia. *PhD thesis*: University of Sydney. [60]	General disability	Thesis; Focus groups and interviews	VI	This thesis identifies and describes the elements influencing participation of Aboriginal people in disability services from the perspectives of both non-Aboriginal and Aboriginal employees in two NSW funded disability services.	No
Higgins, J, & Beecher, S. (2010) The Secretariat of National Aboriginal and Islander Child Care (SNAICC) Early Days Project on Autism Spectrum Disorders August 2010. Victoria: SNAICC. [71]	Autism Spectrum Disorders	Interviews; Case study	VI	The Parenting Research Centre invited SNAICC to help ensure that the Early Days Project on Autism Spectrum Disorders (ASD), a free national program for parents and carers of a child under 6 with an ASD, is culturally appropriate and inclusive of Aboriginal and Torres Strait Islander families.	Yes
McSwan, D., Ruddell, D., Searston, I. (2001). Report: A Whole Community Approach to Otitis Media - reducing its incidence and effects. Townsville: Rural Education, Research & Development Centre, James Cook University. * [50]	Hearing	Evaluation of a feasibility study	VI	Final report of the research project that aimed to reduce the occurrence and impact of OM in 3 Northern Queensland communities, improve learning outcomes for Aboriginal children who have or had OM, and implement culturally appropriate prevention and management practices.	Yes
Ministerial Advisory Committee: Students with Disabilities (2003). Aboriginal Students with Disabilities. South Australia: Government of South Australia. [52]	General disability	Interviews formed into a synopsis of stories; Stakeholder forum	VI	The Ministerial Advisory Committee: Students with Disabilities commenced a project in 2002 to identify issues relating to education for Aboriginal children with a disability to advise the South Australian Minister for Education and Children’s Services on policy directions.	No
Ministerial Advisory Committee: Students with Disabilities (2007). Aboriginal Students with Disabilities: Otitis Media and Conductive Hearing Loss. South Australia: Government of South Australia. [56]	Hearing	Comparative case studies; Interviews; Surveys; Literature review	VI	This study examined the programs established to address the high prevalence of OM and hearing loss experienced by Aboriginal children in urban and regional areas of South Australia.	No
New South Wales Ombudsman (2010). Improving service delivery to Aboriginal people with a disability: a review of the implementation of ADHC's *Aboriginal Policy Framework* and *Aboriginal Consultation Strategy*. Sydney: New South Wales Ombudsman. [73]	General disability	Literature review; Document review; Stakeholder consultations; Interviews; Review of relevant complaints and inquiries	VI	This review examined the Ageing, Disability and Home Care (ADHC) initiatives to achieve the goals of the Aboriginal Policy Framework and Aboriginal Consultation Strategy, and assessed whether they have resulted in better service access for Aboriginal people with a disability and their families.	No
Purcal, C., Newton, BJ., Fisher, KR., Eastman, C., & Mears, T. (2013). School readiness program for Aboriginal children with additional needs: working with children, families, communities and service providers. Interim evaluation report. Sydney: Social Policy Research Centre, UNSW. [62]	General disability	Evaluation using participatory research principles; Literature review; Interviews; Review of program data	VI	This project evaluated the Northcott Disability Services school readiness program for Aboriginal children with additional needs to support their transition to school located in an urban and rural area in New South Wales.	Yes
Queensland Health (2009). Deadly Ears, Deadly Kids, Deadly Communities: 2009–2013. Queensland: Queensland Government. [57]	Hearing	Framework description	VII	Description of the Deadly Ears, Deadly Kids, Deadly Communities: 2009–2013 strategic framework for Queensland to improve the ear health of Aboriginal and Torres Strait Islander children.	No
Scholes, J. (2010). Deadly Ears Speech Pathology: Working through partnerships to limit the impact of otitis media on the communication development of Aboriginal and Torres Strait Islander children. *Talkabout*, 23(2). [36]	Hearing	Discussion paper	VII	Describes the partnership population based approach of Deadly Ears Speech Pathology service within the context of the multidisciplinary Deadly Ears Program.	Yes
Simmons, K., Rotumah, V., Cookson, M., & Grigg, D. (2012). Child Hearing Health Coordinators Tackle Ear and Hearing Health in the NT. *The Chronicle*, 23(1). [69]	Hearing	Program description	VII	Describes the role of the Child Hearing Health Coordinator (CHHC) positions located within the Northern Territory Department of Health, Health Development Unit to coordinate regional programs that are inclusive of hearing health.	No
Western Australia Education and Health Standing Committee (2012). Report on key learnings from the Committee research trip 11–17 March 2012. Perth, WA: Parliament of Western Australia. [54]	Foetal Alcohol Spectrum Disorder; Hearing	Forums; Briefings	VII	Report of a research trip undertaken by the Western Australia Education and Health Standing Committee to explore issues around health and education in North West Western Australia to improve educational outcomes.	No

*Reports on the same study as the included peer-reviewed article by McSwan, D. (2001).

The literature predominantly reported on hearing impairment and related disability, such as learning impairments (n = 17). Of the included articles, 14 provided details on 12 different models involving inter- and intra-sector collaboration. The majority of these models centered on collaboration within different areas of the health sector (intra) (n = 5) and between the health and education sectors (inter) (n = 5). Half of the models (n = 6) were set in schools or early childhood centers and the most common model component (n = 6) was a form of capacity building.

Overall, the qualitative studies were generally well-reported according to Kitto et al’s criteria for assessing qualitative literature [38] that evaluated clarification of research, data collection techniques, justification of qualitative approach, and interpretation. None of the studies reported on whether the sampling techniques supported generalizability and seldom demonstrated transparency of data analysis or researcher reflexivity. The mean STROBE score for the observational studies was 16 out of 22 (73%). Both studies reported well on rationale, study design, setting, variables, data sources, outcome data, and generalizability. Neither study reported on the eligibility/selection of participants, study size or study limitations. The TREND score was 15 out of 22 (68%) for the intervention study, which reported well on background, methods, and results but not generalizability. The mixed method study received a MMAT score of 50% for the qualitative component, reporting well on data sources and relationship between findings and context but not on analysis or researcher influence, 75% for the quantitative component, reporting well on sampling strategy, measurements, and response rates, and 50% for the mixed method component, reporting well on research design but not limitations. The literature review received an AMSTAR score of 78% for the 9 applicable items and reported well on study selection, data extraction, search strategy, study characteristics and quality assessment of studies. The literature review did not provide a list of excluded studies and there was no assessment of publication bias.

The following section provides a narrative synthesis of the findings using the macro- (government), exo- (organizational), and meso- (provider) system levels of the ecological model to demonstrate the components of inter- and intra-sector collaboration in Aboriginal and Torres Strait Islander childhood disability.

### Macro- (government) system factors

#### Factor: Structure of government departments and agencies

The siloed structure of health, education and social service departments and agencies was found to impede service integration and the ability of providers to work collaboratively [48]. Siloes of service provision across government departments and agencies and between levels of government [49] negatively impacts service access for families when they have to navigate different waiting lists and assessment processes, and receive disparate pieces of information from professionals working in isolation [48,50,51]. The fragmentation and complexity of government services [52] impede opportunities for collaboration, with some providers reporting difficulties in locating and communicating with relevant services [52,53]. The adoption of a consultative approach across health, education and social service departments has been recommended as a solution for reducing service duplication and fragmentation and is more aligned with the needs of the child- which are beyond the biomedical and include social, cultural, economic and psychological issues [50].

#### Factor: Policies

Collaboration at the level of policy making can address the barriers generated by existing structures of government departments and agencies. Formalized agreements like memoranda of understanding (MoU) and collaborative frameworks between government sectors can facilitate collaboration at the level of service provision [54]. MoUs between the health and education sectors have promoted collaboration between health professionals and school staff in screening and treatment of middle ear disease to prevent hearing loss [54,55]. Frameworks for whole-of-government approaches have been recognized as important in providing coordinated interagency responses [56–58]. Formalized agreements should focus on detailing a set of long-, medium- and short-term strategies as it provides clarity around collaborative programs for local providers [55,59].

### Exo- (organizational) system factors

#### Factor: Communication - Awareness

Although multiple agencies and services may be involved with the care of a child with a disability, this does not mean that they are all aware of each other’s existence, which can lead to duplication of resources [60]. Both families and providers have identified the lack of communication between, and knowledge of, the different agencies and services as a barrier to accessing available support [53]. Raising awareness of collaborative partnerships through the distribution of educational resources across agencies and services facilitates collaboration and the professional development of providers with little knowledge of disability [52,55,56]. Distribution of these resources helps providers in remote areas of Australia who have reported feeling like they work in isolation [61]. Advertising collaborative projects and the participating personnel also aids collaboration by reducing the risk associated with providers working outside their professional boundaries [50]. Good community awareness of the organization that is providing a program has also been reported to facilitate the establishment of collaborative organizational partnerships with local services [62].

#### Factor: Communication – Lack of role clarity and responsibility

Ambiguity and lack of role clarity and responsibilities of different providers, agencies and organizations is a key barrier to collaboration at the exo- (organizational) system level [57]. The role of Aboriginal Health Workers is unclear to some mainstream providers leading to their underutilisation, despite the important role they play [20]. Formally communicating the role and responsibility of each team member is reported as an essential step when putting into practice an inter-agency or multi-disciplinary model [50].

#### Factor: Financial and human resources

Barriers to the uptake and sustainability of collaborative models include difficulty providing them in sectors that are already facing service provision within a tightening financial environment [48] and a lack of the levels of funding required for providing holistic care approaches [63,64]. Where organizations continue to provide collaborative models of service provision despite lack of appropriate funding they report that this is done so *“on sheer good will”* [63] with staff often working beyond their normal hours [64].

Building effective and trusting collaborative relationships across different organizations, agencies and services takes time [57,62,65]. Collaboration can be impeded when providers lack the time to develop the skills and build the networks required [53].

#### Factor: Service delivery setting

The effectiveness of a collaborative program is influenced by the setting in which it is delivered. Collaboration is facilitated by the delivery of mainstream programs in culturally safe environments for Aboriginal and Torres Strait Islander providers, communities and families [51,53,66]. Delivering collaborative health services within schools has been reported to reduce the stigma and the socio-economic impact of having to attend services in mainstream settings for Aboriginal and Torres Strait Islander families, while increasing program participation [66,67]. Basing health services within schools also allows the services to be responsive to local needs and promotes increased awareness of disability and relevant services among education providers [55,67]. Collaboration between health and education services based in a single setting provides a one-stop-shop, which facilitates the sharing of information between different services and organizations [52].

### Meso- (provider) system factors

A number of key factors of collaboration are found at the front line of collaborative service provision within the meso- (provider) system where the interactions occur between providers, communities and Aboriginal and Torres Strait Islander families and their children.

#### Factor: Relationships

A key facilitator to collaboration at this level is the coordinator or linking role. The appointment of a person external to the services or agencies involved whose role is to link the different players and act as a trainer, motivator and sustainer can be important to a collaborative inter-disciplinary approach [50,68,69]. In the context of Aboriginal and Torres Strait Islander childhood disability, this person is usually local to the community (eg a community liaison person, Aboriginal Education Worker, Aboriginal Health Worker) and is a conduit between providers, communities and families, also promoting the cultural competence of services [52,60,64,66,67,70,71].

The effectiveness of the coordinator or linking role in facilitating collaboration is influenced by the individual’s characteristics. Being open and inclusive and having personal contacts among decision makers in the organizations, agencies, and services involved promotes collaboration [50]. The effect of individual characteristics on collaborative relationships extends to providers. Collaboration can be impeded by specialist providers choosing to only draw knowledge and skills from their traditional disciplines [48]. Aboriginal and Torres Strait Islander provider experiences of racism and historical trauma can obstruct engagement with mainstream services [53]. Awareness of cultural difference and individual attitudes [72] and getting along well with people [66] are individual provider characteristics that can facilitate collaborative relationships. Transience and turnover of key staff can disrupt collaborative efforts [50,56,68].

Building relationships integral to collaboration at the local level is facilitated by face-to-face provider engagement and ‘linking’ with communities [48,58,73]. Provider-to-provider engagement is facilitated by demonstrating mutual respect and understanding [50,72], having access to direct links for communication, and using open and respectful communication strategies [50,51]. The importance of engagement is reflected in the collaborative Specialist Integrated Community Engagement (SpICE) model that is based around the concept of ‘linking’ different sectors and the community through engagement to build social capital and a ‘community of learners’ to sustain the collaborative process [48]. Engaging the community can be important to the success of collaborative programs [74] and tapping into existing collaborative relationships in the community can facilitate the engagement process [67]. Where a mainstream organization is unknown to a community, attending interagency meetings in the local area by their providers can facilitate engagement with Aboriginal and Torres Strait Islander organizations [62].

#### Factor: Inter- and intra-professional learning

The modeling of inter- and intra-professional collaboration by clinical educators from different disciplines for university students on placement has been reported to facilitate a well-coordinated and holistic approach to learning [72]. The sustainability of collaborative practices is increased by empowering students to incorporate the lessons learned into their future practice [72]. Inter- and intra-professional learning also facilitates collaboration by creating supportive relationships between providers from different disciplines [66].

## Discussion

The findings of this review depict a national snapshot of collaboration addressing the limited understanding of how collaboration works in practice in the field of Aboriginal and Torres Strait Islander childhood disability. The complex nature of childhood development, particularly for Aboriginal and Torres Strait Islander children, has seen recognition of the need for a shift from a purely medical view of disability to collaborative approaches that also take into account social and environmental factors [47,48,53]. Divisions between mainstream, specialist and non-mainstream services can result from top-down approaches that do not work for addressing complex problems which require ‘buy-in’ to collaborative approaches at all levels [30,75]. In the move towards collaboration, however, it is important to recognize that collaboration is, in itself, a complex concept which has the potential to inspire innovative solutions or create frustration [76]. Further research is required into collaborations in Aboriginal and Torres Strait Islander childhood disability to maximize the potential, and minimize any negative impacts, of collaborative approaches. The paucity of research on Aboriginal and Torres Strait Islander children with a disability [4] also means exploring the experiences of children and their families in accessing services is important to completing a holistic picture in order to improve service access.

The importance of respectful communication and culturally appropriate program delivery as found in this review demonstrates the need for cultural competence as a central pillar of collaboration in Aboriginal and Torres Strait Islander childhood disability. Cultural competence requires promotion of attitudes, knowledge and behavior at individual, institutional and systemic levels in order to deliver effective care for Aboriginal and Torres Strait Islander peoples [77]. Culturally competent organizations and systems need to be reflective of the diverse populations they serve, including at leadership and management levels, and through policies which facilitate cross-cultural communication and access [78]. An increased focus on cultural competence may help to address the negative impact of racism on service access and provision.

Although the review focused on Aboriginal and Torres Strait Islander children and is not necessarily generalizable to other indigenous populations, similar health disparities are experienced by indigenous populations worldwide [9,10,47,79]. Investment of time as a facilitator to building sustainable collaborations in the face of government policy and funding cycles is reflected in Canada’s collaborative Aboriginal Head Start program to improve indigenous child development outcomes. A key element to the positive impact of the community-based program is the time it took (more than a decade) to establish credibility within communities and build a trained and experienced workforce to work collaboratively [9]. Long-term commitment to sustainable and collaborative relationships with indigenous organizations and communities is also a strategy identified by Aboriginal and Torres Strait Islander organizations to achieve genuine partnerships [80].

Building workforce capacity has been recommended as a key element in improving service access for people with a disability and addressing the social determinants of health [8,47]. Health providers, in particular, have been identified as key players through advocacy, working in partnerships, and working with communities [81]. Collaboration is more likely to be achieved through personal relationships than imposed structures [82], further emphasizing the important role of health, education, and social service providers in improving service access for Aboriginal and Torres Strait Islander childhood disability through collaboration.

### Limitations

The conclusions of systematic reviews are inevitably limited by the breadth and quality of the research available for inclusion. Literature relevant to the topic of interest has been mostly discursive, with only eight empirical studies published in a peer-review journal, only one of which has tested an intervention. The focus of the review on Aboriginal and Torres Strait Islander children with a disability across Australia may mean that it is not generalizable to indigenous populations in other countries or to specific Aboriginal and Torres Strait Islander populations within Australia. This review provides a broad national snapshot of collaboration, but further research within specific local contexts is required to explore ways collaboration can improve access and be responsive to local needs [8,80]. Due to the focus of the review on inter- and intra-sector collaboration, no data for the microsystem of the family and the individual child were collected. The intra- and inter-personal factors and interactions at this level, however, both influence and are influenced by the factors of collaboration at the meso- (provider), exo- (organizational) and macro- (government) system levels.

## Conclusions

The policy shift towards inter-sector collaborative approaches represents a strong opportunity for the health, education, and social service sectors and their providers to work collaboratively with each other in innovative ways. As this review has shown however, collaboration is not a simple concept. Many barriers and facilitators exist at the macro- (government), exo- (organizational) and meso- (provider) system levels that influence the effectiveness of collaborative efforts. By identifying the components of inter- and intra-sector collaborations this review provides information to guide future efforts at developing collaborative solutions to improve service access for Aboriginal and Torres Strait Islander children with a disability and their families.

## Abbreviations

OM: Otitis Media
WHO: World Health Organization
MoU: memoranda of understanding
MeSH: Medical Subject Headings
CINAHL: Cumulative Index to Nursing and Allied Health Literature
ERIC: Education Resources Information Center
APAIS: Australian Public Affairs Information Service
APAIS-health: Australian Public Affairs Information Service - Health
A&TSIhealth: Aboriginal and Torres Strait Islander Health
MAIS-ATSIS: Multicultural Australia and Immigration Studies - Aboriginal and Torres Strait Islander Subset
STROBE: STrengthening the Reporting of OBservational studies in Epidemiology
TREND: Transparent Reporting of Evaluations with Nonrandomized Designs
MMAT: Mixed Methods Appraisal Tool
AMSTAR: Measurement Tool to Assess Systematic Reviews
SpICE: Specialist Integrated Community Engagement
